# Effectiveness and Safety of Switching to Ravulizumab From Eculizumab in Kidney Transplant Recipients With Atypical Hemolytic Uremic Syndrome: A Global aHUS Registry Analysis

**DOI:** 10.1111/ctr.70278

**Published:** 2025-08-28

**Authors:** Anja Gaeckler, Imad Al‐Dakkak, Nuria Saval, Hans Herman Dieperink, Margriet Eygenraam, Larry A. Greenbaum, Nicole Isbel, Johan Vande Walle

**Affiliations:** ^1^ Department of Nephrology University Hospital Essen University Duisburg‐Essen Essen Germany; ^2^ Global Medical Affairs Alexion, AstraZeneca Rare Disease Boston Massachusetts USA; ^3^ Department of Nephrology Odense University Hospital Odense Denmark; ^4^ aHUS Canada Brampton Ontario Canada; ^5^ Division of Pediatric Nephrology Emory University School of Medicine and Children's Healthcare of Atlanta Atlanta Georgia USA; ^6^ Department of Kidney and Transplant Services The University of Queensland and Princess Alexandra Hospital Brisbane Queensland Australia; ^7^ Department of Pediatrics and Medical Genetics Ghent University Hospital Ghent Belgium

**Keywords:** atypical hemolytic uremic syndrome, complement, global aHUS registry, kidney transplantation, ravulizumab, real‐world data, thrombotic microangiopathy

## Abstract

**Introduction:**

Atypical hemolytic uremic syndrome (aHUS) is a disease of complement dysregulation that may lead to kidney failure. Ravulizumab and eculizumab are complement C5 inhibitors approved for the treatment of aHUS. This study assessed the real‐world effectiveness and safety of switching to ravulizumab from eculizumab in kidney transplant recipients with aHUS.

**Methods:**

The Global aHUS Registry is a multicenter study enrolling patients with aHUS since 2012. Effectiveness and safety outcomes were assessed in kidney transplant recipients with aHUS who switched to ravulizumab from eculizumab up to September 2, 2024.

**Results:**

Overall, 38 patients received a kidney transplant before ravulizumab initiation; 27 patients with ≥3 months of ravulizumab treatment were included in the patient characteristics and effectiveness analyses. Median (range) time on eculizumab and ravulizumab treatment was 66.1 (3.7, 158.3) and 24.1 (4.2, 49.3) months, respectively (*n* = 27); time from last kidney transplantation to ravulizumab initiation was 65.9 (3.7, 184.0) months. Following ravulizumab initiation, laboratory parameters remained stable, and no kidney transplant rejections/graft failures were reported. In the safety analysis (*n* = 38), 23 adverse events were reported in 19 patients (50.0%) at or after ravulizumab initiation, and none were considered treatment‐related. No new events of thrombotic microangiopathy or kidney impairment and no meningococcal infections or deaths were reported.

**Conclusion:**

This analysis from the Global aHUS Registry provides real‐world evidence to demonstrate that the transition to ravulizumab from eculizumab in kidney transplant recipients with aHUS is successful, with stable graft function with no treatment‐related safety concerns.

**Trial Registration:**

ClinicalTrials.gov identifier: NCT01522183

**Abbreviations**
AE: adverse event
aHUS: atypical hemolytic uremic syndrome
eGFR: estimated glomerular filtration rate
KDIGO: Kidney Disease: Improving Global Outcomes
LDH: lactate dehydrogenase
TMA: thrombotic microangiopathy

## 1 Introduction

Atypical hemolytic uremic syndrome (aHUS) is a progressive, rare disease that, if untreated, can result in severe organ damage and death [1]. aHUS is a form of thrombotic microangiopathy (TMA) caused by complement dysregulation that leads to uncontrolled terminal complement activation that can manifest in the presence or absence of a trigger or associated condition [1, 2, 3].

Eculizumab is a humanized monoclonal antibody that has changed the natural history of aHUS; eculizumab acts by blocking terminal complement activation through inhibiting cleavage of complement C5 and was approved for the treatment of aHUS in 2011 [2, 3, 4, 5]. Ravulizumab is a next‐generation terminal complement inhibitor derived from eculizumab with a longer half‐life and has been approved for the treatment of aHUS (among other indications) in the United States (2019), Europe (2020), Japan (2020), and other regions [6, 7, 8, 9]. Ravulizumab has demonstrated immediate, complete, and sustained terminal complement inhibition in clinical trials and can be administered once every 4–8 weeks using individualized weight‐based dosing [10, 11, 12, 13]. In patients with aHUS, ravulizumab resolves TMA and improves kidney function, platelet counts, and markers of hemolysis. Sustained treatment with ravulizumab over 2 years achieves continued stabilization and/or improvement in these parameters [14].

Kidney impairment is recognized as a severe complication of aHUS, and a substantial proportion of patients with aHUS progress to kidney failure, either before or after kidney transplantation [15, 16, 17]. Graft endothelium damage may occur following kidney transplantation owing to an increase in complement activity post‐transplantation, which predisposes to the recurrence of TMA in susceptible patients. Additional factors that may contribute to endothelium damage include alloimmune responses, ischemia‐reperfusion events, immunosuppressive drugs, and viral infections [18, 19].

In the absence of complement inhibitor treatment, kidney transplantation in aHUS is associated with high rates of TMA recurrence post‐transplantation, leading to early graft failure [20]. The risk of disease recurrence post‐transplantation is especially high in kidney transplant recipients with aHUS due to pathogenic variants in *CFH*, *C3*, or *CFB*, with a risk of relapse ranging from 45% to 100%, although disease recurrence may occur in the absence of these variants [21, 22, 23]. Notably, before the approval of eculizumab, kidney transplantation was contraindicated in patients at high risk of disease recurrence [18]. However, in a previous study of 22 patients with a kidney transplant, of whom nine had a genetic complement abnormality associated with high risk of aHUS recurrence, treatment with eculizumab successfully prevented disease recurrence post‐transplantation in 8/9 patients [24]. Guidelines recommend prophylactic eculizumab treatment for patients with moderate or high risk for disease recurrence after kidney transplantation [2, 25]. Additionally, eculizumab treatment should also be considered in patients with de novo aHUS triggered by prior kidney transplantation [23, 26, 27].

Although real‐world evidence on the safety and effectiveness of switching to ravulizumab from eculizumab in patients with aHUS has been previously described, studies have not specifically analyzed outcomes in kidney transplant recipients switching treatments [28, 29]. We therefore assessed the real‐world clinical characteristics and outcomes of kidney transplant recipients with aHUS who switched to ravulizumab from eculizumab using data from the Global aHUS Registry.

## 2 Methods

### 2.1 Study Design

The Global aHUS Registry is a multicenter study, sponsored by Alexion Pharmaceuticals Inc., collecting both prospective and retrospective data on demographics, characteristics, natural history, and treatment outcomes in patients with aHUS who provided written informed consent before study participation [30]. Additional study design details were described previously [28].

Adult and pediatric kidney transplant recipients with aHUS who switched to ravulizumab from eculizumab up until the data‐cut of September 2, 2024 were included in this analysis. The safety analysis population for the current study included all kidney transplant recipients with aHUS who switched to ravulizumab from eculizumab. The efficacy analysis included all patients with aHUS who switched to ravulizumab from eculizumab with more than 3 months of ravulizumab treatment and up to 1 month between eculizumab discontinuation and ravulizumab initiation, with a ravulizumab initiation date on or after October 1, 2019. These criteria ensured that patients had a clinically meaningful ravulizumab treatment duration and excluded patients who discontinued eculizumab, relapsed, and then initiated treatment with ravulizumab. Patients may have discontinued ravulizumab prior to the last follow‐up.

### 2.2 Patient Characteristics

The patient characteristics extracted for analysis included age at ravulizumab initiation, age at kidney transplantation, sex, time on ravulizumab and eculizumab treatment, time from aHUS onset to eculizumab and ravulizumab initiation, eculizumab and ravulizumab treatment duration, complement gene variants, anti‐complement factor H (CFH) antibody status, and time from last kidney transplantation to ravulizumab initiation.

### 2.3 Efficacy and Safety Outcomes

Clinical events, assessed as part of the effectiveness outcomes, included the number of kidney transplantation events before ravulizumab initiation, time from last kidney transplantation to ravulizumab initiation, number of kidney transplant failures/rejections, time from ravulizumab initiation to kidney transplant failure/rejection, and reported TMA signs or symptoms. Laboratory parameters assessed included estimated glomerular filtration rate (eGFR; calculated using the Chronic Kidney Disease Epidemiology Collaboration method for adult patients [normal range for adults: >90 mL/min/1.73 m^2^] and the Schwartz method for pediatric patients), creatinine level (normal range for adults: male, 65.4–119.3 µmol/L; female, 52.2–91.9 µmol/L), LDH level (normal range for adults: 120–220 U/L), and platelet count (normal range: 150 × 10^9^/L to 450 × 10^9^/L) were also assessed. The safety analysis included number of adverse events (AEs), meningococcal infections, and deaths, reported on or after ravulizumab initiation.

### 2.4 Statistical Analysis

This was a descriptive study. Continuous data were summarized as median (range) and categorical data were summarized as number and percentage of patients. Laboratory parameters were presented as median (interquartile range [IQR]).

## 3 Results

### 3.1 Demographic and Clinical Characteristics

Overall, 38 patients (33 adults; 5 pediatrics) received a kidney transplant prior to switching to ravulizumab and were included in the safety analyses (Figure S1). Of those, 27 patients (24 adults; 3 pediatrics) were included in the main analysis population for patient characteristics and effectiveness (11 patients were excluded, including nine with less than 3 months of ravulizumab treatment and two with more than 1 month between eculizumab discontinuation and ravulizumab initiation).

Patients in the main analysis population (*n* = 27) had a median (range) age of 35.8 (10.9, 72.9) years at ravulizumab initiation, 32.5 (4.0, 68.1) years at kidney transplantation, and the majority were female (16/27 [59.3%]; Table 1). Median (range) time on treatment was 66.1 (3.7, 158.3) months for eculizumab and 24.1 (4.2, 49.3) months for ravulizumab and time from last kidney transplantation to ravulizumab initiation was 65.9 (3.7, 184.0) months.

**TABLE 1:** Patient disposition and baseline characteristics (main analysis population; *n* = 27).

	Pediatric (*n* = 3)	Adult (*n* = 24)	All patients (*n* = 27)
Age at ravulizumab initiation, years, median (range)	11.3 (10.9, 14.0)	37.8 (26.4, 72.9)	35.8 (10.9, 72.9)
Age at kidney transplant, years, median (range)	4.6 (4.0, 5.5)	33.8 (11.0, 68.1)	32.5 (4.0, 68.1)
Sex, *n* (%)			
Female	0	16 (66.7)	16 (59.3)
Male	3 (100)	8 (33.3)	11 (40.7)
Time on ravulizumab treatment, months, median (range)	18.9 (16.4, 49.3)	24.3 (4.2, 47.0)	24.1 (4.2, 49.3)
Time on eculizumab treatment, months, median (range)	107.4 (96.0, 113.9)	65.9 (3.7, 158.3)	66.1 (3.7, 158.3)
Genetic status, *n* (%)			
Any pathogenic variant found^a^	0	14 (58.3)	14 (51.9)
*CFH*	0	6 (25.0)	6 (22.2)
*C3*	0	4 (16.7)	4 (14.8)
*CFI*	0	4 (16.7)	4 (14.8)
*CD46* (MCP)	0	2 (8.3)	2 (7.4)
Thrombomodulin	0	1 (4.2)	1 (3.7)
Anti CFH‐antibody tested and positive	0	3 (12.5)	3 (11.1)
Any pathogenic variant found or anti‐CFH antibody positive	0	15 (62.5)	15 (55.6)
Total number of kidney transplants, *n* ^b^	3	32	35
Time from last kidney transplant to ravulizumab initiation, months, median (range)	82.7 (69.7, 113.9)	59.8 (3.7, 184.0)	65.9 (3.7, 184.0)

Abbreviations: C3, complement C3; CD46, cluster of differentiation 46; CFH, complement factor H; CFI, complement factor I; MCP, membrane cofactor protein.

^a^ Patients may have had more than one pathogenic variant. Patients were also tested for complement factor B mutations, but no patients with variants were identified.

^b^ For patients with available data (*n* = 10), eight kidney transplants were from deceased donors and two from live donors.

### 3.2 Genetic Analyses

In total, 17/27 patients (63.0%) were tested for pathogenic variants in at least five complement genes. Pathogenic variants in complement genes were reported in 14/27 patients (51.9%; Table 1) and the most common pathogenic variant was in *CFH* (detected in 6/14 patients [42.9%]). There were 18/27 patients (66.7%) who were tested for anti‐CFH antibodies and three were positive (11.1%); none of these three patients received immunosuppression specifically targeting anti‐CFH antibodies (e.g., rituximab).

### 3.3 Clinical Events

**FIGURE 1:**
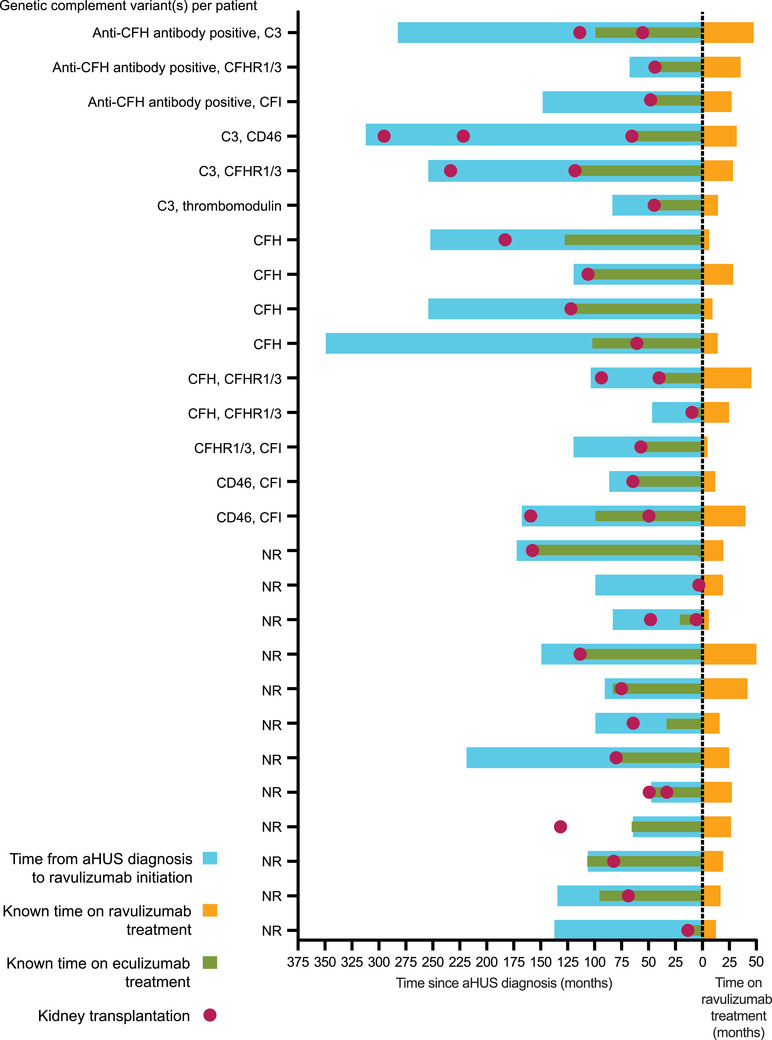
Kidney transplantation events since diagnosis of aHUS (main analysis population; *n* = 27). aHUS, atypical hemolytic uremic syndrome; C3, complement C3; CFH, complement factor H; CFI, complement factor I; CFHR1/3, complement factor H related 1/3; NR, not reported.

Prior to ravulizumab initiation, there were 35 kidney transplantations among the 27 patients in the main analysis (Figure 1). Of those, 23 transplantations were reported in 20 patients at or before eculizumab initiation (up to a maximum 229.7 months before eculizumab initiation), and 12 kidney transplantation events in 11 patients were reported after eculizumab initiation (ranging from 8.7 to 77.6 months post‐eculizumab initiation). Notably, all kidney transplantations occurred prior to switching to ravulizumab.

Overall, time from any kidney transplantation to ravulizumab initiation ranged from 3.7 to 295.7 months, with a median (IQR) of 66.5 (48.8, 116.5) months. No events of kidney transplantation or TMA signs or symptoms were reported after ravulizumab initiation with a median ravulizumab treatment duration of 24.1 months. Additionally, no kidney transplant rejections or failures were reported after ravulizumab initiation and no patient received dialysis while on ravulizumab.

Two patients had a kidney transplant in the 6 months before switching to ravulizumab from eculizumab. For one of these patients, eculizumab treatment was initiated at the same time as kidney transplantation and the patient remained on eculizumab for 3.8 months before switching to ravulizumab. In the second patient, two kidney transplantations at 6.0 and 48.8 months prior to ravulizumab initiation and during eculizumab treatment were reported. Additionally, one patient with a PAX2 mutation (that can be associated with kidney hypodysplasia) received one kidney transplantation 66 months prior to diagnosis of aHUS. In total, six patients received two successive kidney transplants prior to or during eculizumab treatment with time between transplantations ranging from 15 to 115 months. Further, one patient received three successive kidney transplants; 73 months elapsed between the first and second transplantation (prior to eculizumab initiation) and 156 months elapsed between the second and third transplantation (the patient initiated eculizumab at the same time as receiving the third transplant).

### 3.4 Changes in Laboratory Parameters Over Time

**FIGURE 2:**
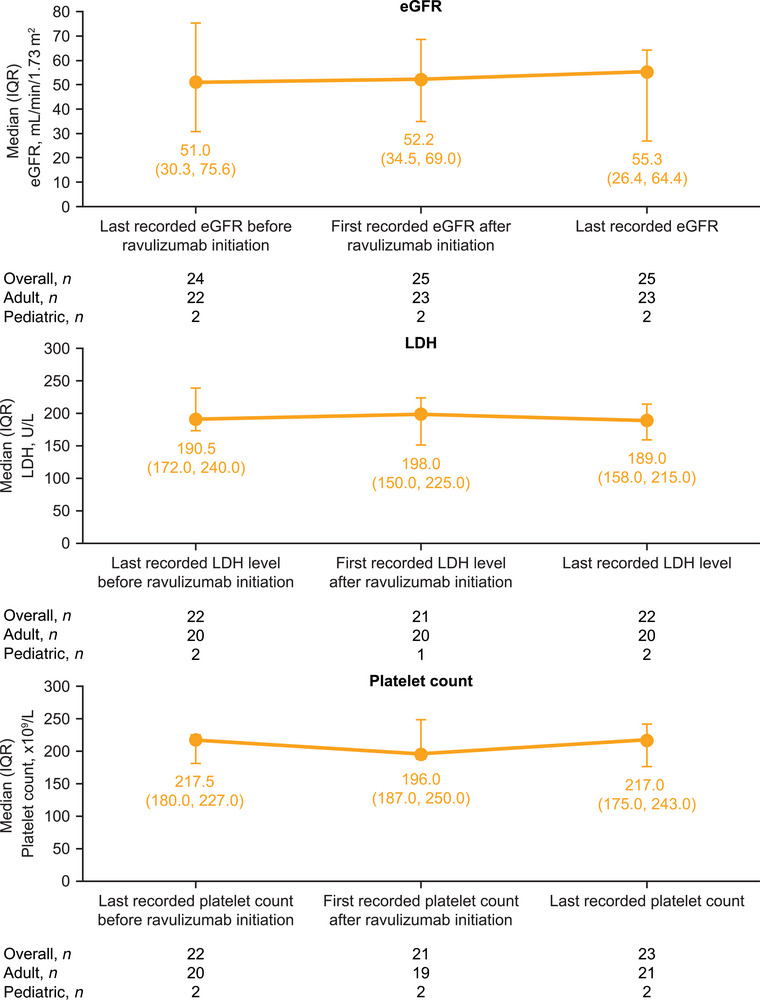
Laboratory parameters (main analysis population; *n* = 27). Patients with available data at each time point are shown. eGFR, estimated glomerular filtration rate; IQR, interquartile range; LDH, lactate dehydrogenase.

eGFR, creatinine, LDH, and platelet count remained stable following the switch from eculizumab and during subsequent treatment with ravulizumab (Figures 2 and S2). For eGFR, the median (IQR) was 51.0 (30.3, 75.6) mL/min/1.73 m^2^ at last record prior to or at ravulizumab initiation (*n* = 24), 52.2 (34.5, 69.0) mL/min/1.73 m^2^ after ravulizumab initiation (*n* = 25), and 55.3 (26.4, 64.4) mL/min/1.73 m^2^ at last follow‐up (*n* = 25).

### 3.5 Safety

In the safety analysis set (*N* = 38), 23 AEs were reported in 19 patients at or after ravulizumab initiation (Table 2). The most common AEs were infection, reported in 10 patients (12 events: two events of SARS‐CoV‐2 infection, two events in one patient of gastroenteritis, and one event each of influenza A viral infection, *Escherichia coli* infection in the blood, kidney transplant pyelonephritis, norovirus infection, pulmonary nodular infiltration, pneumonia, urosepsis, and sepsis) and malignancy (three events: squamous cell carcinoma, malignancy [undefined], and lymphoproliferative syndrome [Burkitt lymphoma]). No meningococcal infections or deaths were reported while on ravulizumab and no AE was considered related to ravulizumab treatment.

**TABLE 2:** Safety outcomes (safety analysis population; *N* = 38).

Adverse event	Number of patients with an adverse event, *n* (%)
Infection^a^	10 (26.3)
Malignancy^b^	3 (7.9)
Anemia	1 (2.6)
Atrial fibrillation	1 (2.6)
Disseminated intravascular coagulation	1 (2.6)
Hip pain	1 (2.6)
Hospitalization due to elevated potassium	1 (2.6)
Seizure	1 (2.6)
Sigmoid colon perforation	1 (2.6)
Superior vena cava thrombosis	1 (2.6)

*Note:* Patients may have had more than one adverse event.

^a^ Two events of SARS‐CoV‐2 infection, two events of gastroenteritis, and one event of influenza A viral infection in one patient, and one event each of *Escherichia coli* infection in the blood, kidney transplant pyelonephritis, norovirus infection, pulmonary nodular infiltration, pneumonia, urosepsis, and sepsis.

^b^ One event each of squamous cell carcinoma, malignancy (undefined), and lymphoproliferative syndrome (Burkitt lymphoma).

Most infections during ravulizumab administration (9/12, 75%) were considered by the investigator as mild or moderate in severity; 11/12 (92%) required hospitalization or prolongation of hospitalization. During eculizumab administration, 29 AEs of infection were reported in seven patients (with 19 of these events occurring in two patients); most were considered mild or moderate (24/29, 83%) and 19/29 (66%) required hospitalization or prolongation of hospitalization.

## 4 Discussion

This study provides real‐world data on the safety and efficacy of switching to ravulizumab from eculizumab in kidney transplant recipients with aHUS, a particularly vulnerable population with increased risk of disease recurrence post‐transplantation.

This analysis used data from the Global aHUS Registry and reports the longest real‐world treatment exposure and follow‐up data to date. During ravulizumab treatment, kidney function and hematological parameters remained stable, and no rejection events or graft failures were reported after switching to ravulizumab from eculizumab. Additionally, no meningococcal infections or deaths were reported, and no AE was unexpected or considered treatment‐related, which is consistent with the favorable safety profile of ravulizumab established in previous clinical trials [10, 11, 12, 13].

The efficacy and safety results reported in this study are in line with a previously published real‐world evidence study that included 10 kidney transplant recipients with aHUS who switched to ravulizumab after 3 months of eculizumab treatment and reported stable hematological parameters with no clinical signs of TMA relapse [29]. Case reports have also shown favorable outcomes [31, 32, 33]. Currently, there are no clinical trials reporting safety and efficacy in kidney transplant recipients with aHUS who switched to ravulizumab from eculizumab. Nevertheless, a previous phase 3 trial of ravulizumab in patients with aHUS naive to complement C5 inhibitor treatment included eight patients who received a kidney transplant prior to ravulizumab initiation [11]. Of those, three required ravulizumab treatment for longer than 26 weeks to achieve complete TMA response (defined as normalization of platelet counts [≥150 × 10^9^/L] and levels of lactate dehydrogenase [LDH; ≤246 U/L], and ≥25% improvement in serum creatinine) [11], potentially indicating the need for long‐term ravulizumab treatment in kidney transplant recipients with aHUS.

The need for long‐term monitoring and treatment of patients with aHUS post‐transplantation with eculizumab is documented. Guidance from the Kidney Disease: Improving Global Outcomes (KDIGO) conference in 2017 advised caution in considering eculizumab cessation in kidney transplant recipients, particularly in the context of previous allograft loss [2], and a more recent report in 2023 highlighted the need for individualized management of aHUS (including duration of treatment and treatment interruption) [34]. Results from this analysis support the successful transition to ravulizumab from eculizumab, which may guide recommendations and/or considerations for the management of kidney transplant recipients with aHUS. Although the reasons for patients with a kidney transplant switching from eculizumab to ravulizumab were not captured in the Registry, other studies reported that reduced infusion frequency [35] or reduced healthcare resource utilization were among the factors associated with ravulizumab treatment preference over eculizumab in patients with aHUS [27]. In a real‐world database study of healthcare resource utilization, patients reported fewer claims for outpatient, private practice, and home visits, and lower healthcare costs 3–6 months after switching from eculizumab to ravulizumab [36].

Ravulizumab may improve patient health‐related quality of life (HRQoL) compared with eculizumab owing to its reduced infusion frequency and requirement to visit a healthcare center [31, 35]; this added value is particularly important in kidney transplant patients who may require long‐term ravulizumab treatment [37]. In a prior retrospective case series of three pediatric patients with aHUS, ravulizumab maintained remission of aHUS during kidney transplantation and permitted sufficiently reduced frequency of infusion to allow for administration by peripheral cannulation (long‐term central vascular access is often required for eculizumab delivery, especially in children) [37]. Additionally, in a case series of two patients with aHUS who transitioned to ravulizumab from eculizumab after kidney transplantation owing to difficulty in finding venous access for eculizumab and concerns over treatment compliance, respectively, patient‐reported HRQoL assessed via the Functional Assessment of Chronic Illness Therapy (FACIT)‐Fatigue scale showed improvements in social and emotional well‐being within the first 6 months after transition [31]. Finally, in a survey of 50 adults and 16 caregivers of pediatric patients with aHUS, most adult patients (94.0%) and all caregivers reported an overall preference for ravulizumab over eculizumab, with infusion frequency being one of the main factors for patients when selecting their preferred treatment [35]. In the survey, fewer patients reported disruption to daily life and/or to the ability to go to work/school due to ravulizumab infusion frequency (4.0% and 5.7%, respectively) than eculizumab (72.0% and 60.0%), with similar results for caregivers [35]. In the United Kingdom, a report from the National Institute for Health and Clinical Excellence recommended ravulizumab as a cost‐effective treatment option for patients with aHUS who were naive to C5 inhibitor therapy or had responded to ≥3 months of treatment with eculizumab [38]. In a modeling study of patients with aHUS in Germany, Italy, the United Kingdom, and the United States, ravulizumab was estimated to provide a reduction in aggregate duration of treatment of 44%–74%, reduced lost productivity costs due to treatment by 56%–76%, and reduced overall discounted annual treatment costs compared with eculizumab [39].

In this analysis, most patients with aHUS who received eculizumab were treated prophylactically or during kidney transplantation. Prophylactic eculizumab treatment is recommended by KDIGO for patients with aHUS at high risk of disease occurrence, including those with pathogenic mutations and/or previous early recurrence. For patients with moderate risk of recurrence, prophylactic eculizumab can be used at the discretion of the clinician [2]. In a retrospective study of patients with aHUS in France, prophylactic use of eculizumab significantly reduced risk of disease recurrence post‐transplantation, and was associated with longer graft survival [40]; these findings have been confirmed by several other studies [41, 42, 43, 44, 45, 46]. Evidence of prophylactic use of ravulizumab in patients with aHUS is limited to case reports; however, there were no clinical signs of TMA recurrence following kidney transplantation, and kidney parameters remained stable [47, 48]. Studies in larger patient populations are required to confirm these findings.

The limitations of this study include missing data and variable length of patient follow‐up, which are inherent to Registry‐derived data. Patients included in the study were from a subset of countries where ravulizumab was available and there were few pediatric patients eligible for analysis, which limits the generalizability of the results. Additionally, not all patients had a complete genetic evaluation and were screened for anti‐CFH antibodies to confirm a diagnosis of aHUS. The severity of an infection‐related AE was not formally captured in the Registry but was recorded at the discretion of the investigator and hence these data may not be comprehensive. The role of eculizumab or ravulizumab in these infections is uncertain given that infections are a common cause of morbidity and mortality in kidney transplant recipients [49]. Also, the Registry did not include data on pharmacokinetics or pharmacodynamics of ravulizumab, or any measurement of complement C5 or membrane attack complex levels. Nevertheless, findings from observational studies can complement findings from interventional clinical trials and provide real‐world information on long‐term outcomes of safety and effectiveness. In addition, observational registry studies obtain natural disease or treatment response data over time that would otherwise be limited if performed by individual centers. Last, treatment duration was guided by the treating physician rather than by a study protocol; future studies should consider examining if long‐term terminal complement C5 inhibitor treatment is necessary.

In conclusion, this analysis from the Global aHUS Registry provides real‐world evidence of the successful transition from eculizumab to ravulizumab in kidney transplant recipients with aHUS, in whom the disease can be particularly severe with a high chance of disease recurrence. Overall, patients demonstrated stable kidney function and there were no treatment‐related AEs, meningococcal infections, or deaths reported during treatment with ravulizumab.

## Author Contributions

All authors provided substantial contributions to the conception or design of the work; or the acquisition, analysis, or interpretation of data for the work; and drafted the work or revised it critically for important intellectual content; and provided final approval of the version to be published; and agreed to be accountable for all aspects of the work in ensuring that questions related to the accuracy or integrity of any part of the work are appropriately investigated and resolved.

## Conflicts of Interest

Anja Gaeckler and Larry A. Greenbaum have received consultant and lecture fees from, and were scientific advisory board members for, Alexion, AstraZeneca Rare Disease. Imad Al‐Dakkak was an employee of Alexion, AstraZeneca Rare Disease at the time of the study. Nuria Saval is an employee of Alexion, AstraZeneca Rare Disease. Hans Herman Dieperink has nothing to disclose. Margriet Eygenraam has received an honorarium from, and is a scientific advisory board member for, Alexion, AstraZeneca Rare Disease. Nicole Isbel and Johan Vande Walle have received honoraria from, and were scientific advisory board members for, Alexion, AstraZeneca Rare Disease.

## Supporting information




**FIGURE S1:** Patient disposition. **FIGURE S2:** Serum creatinine levels before and after ravulizumab initiation (main analysis population; n = 27).

## Data Availability

Alexion, AstraZeneca Rare Disease will consider requests for disclosure of clinical study participant‐level data provided that participant privacy is assured through methods like data de‐identification, pseudonymization, or anonymization (as required by applicable law), and if such disclosure was included in the relevant study informed consent form or similar documentation. Qualified academic investigators may request participant‐level clinical data and supporting documents (statistical analysis plan and protocol) pertaining to Alexion‐sponsored studies. Further details regarding data availability and instructions for requesting information are available in the Alexion Clinical Trials Disclosure and Transparency Policy at https://www.alexionclinicaltrialtransparency.com/data‐requests/
