# Supplementary material for: Effectiveness and Safety of Switching to Ravulizumab From Eculizumab in Kidney Transplant Recipients With Atypical Hemolytic Uremic Syndrome: A Global aHUS Registry Analysis
Source: Clin Transplant. 2025 Aug 28;39(9):e70278. doi: 10.1111/ctr.70278 (PMC12393192; doi:10.1111/ctr.70278)
Supplement: Supplementary file 1 — FIGURE S1: Patient disposition. FIGURE S2: Serum creatinine levels before and after ravulizumab initiation (main analysis population; n = 27). [file CTR-39-e70278-s001.docx]

Anja Gaeckler, Imad Al-Dakkak, Nuria Saval, Hans Herman Dieperink, Margriet Eygenraam, Larry A. Greenbaum, Nicole Isbel, Johan Vande Walle

Effectiveness and Safety of Switching to Ravulizumab from Eculizumab in Kidney Transplant Recipients with Atypical Hemolytic Uremic Syndrome: A Global aHUS Registry Analysis

# **Supplemental Information**

**FIGURE S1. Patient disposition.**


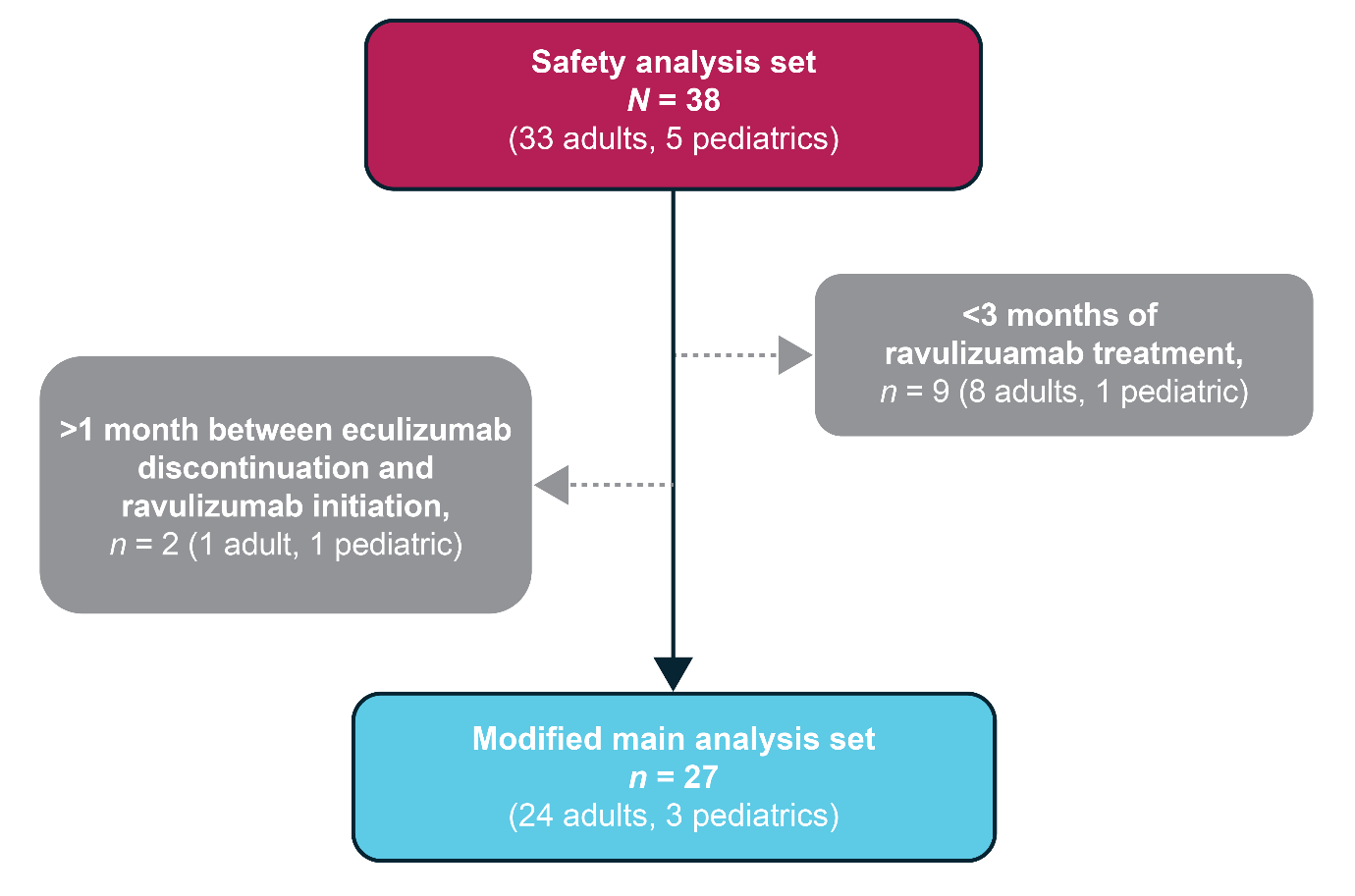


**FIGURE S2. Serum creatinine levels before and after ravulizumab initiation (main analysis population; *n* = 27).**


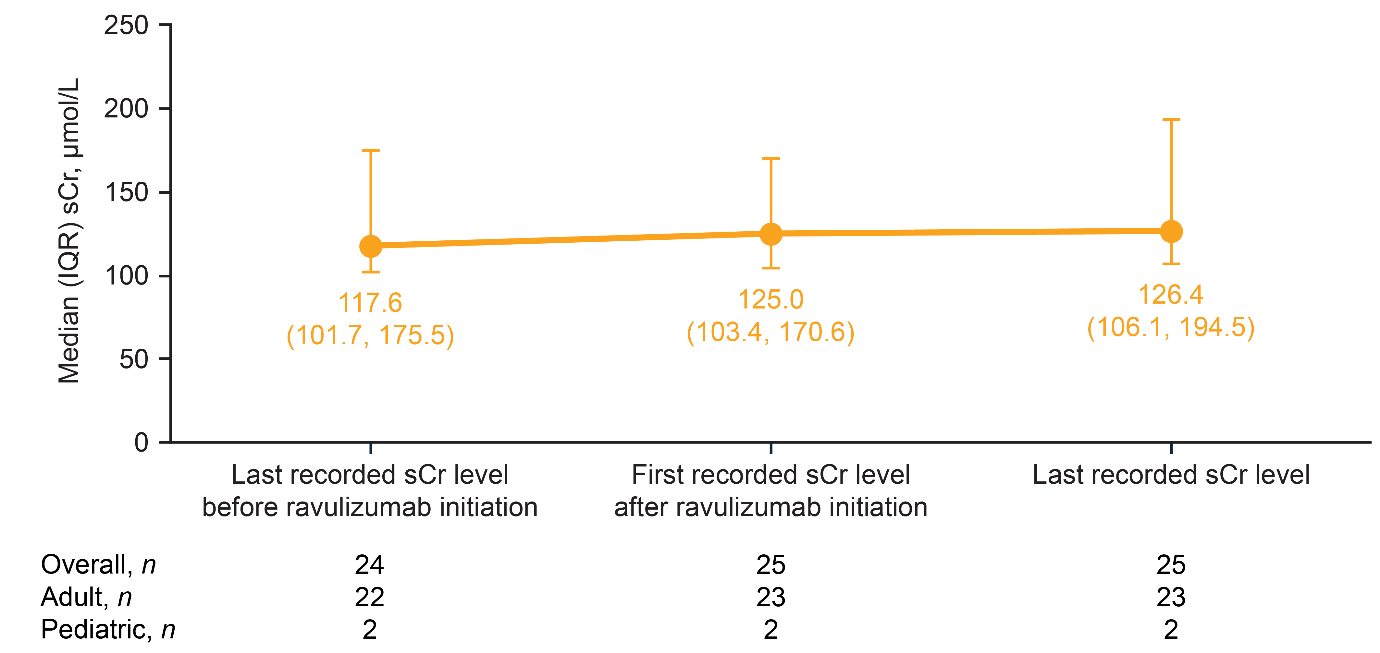


IQR, interquartile range; sCr, serum creatinine.

Patients with available data at each time point are shown.
